# Anticipated Social and Healthcare Economic Burden of People with Alzheimer’s Disease in Two Selected Regions of the Czech Republic

**DOI:** 10.3390/healthcare8040433

**Published:** 2020-10-26

**Authors:** Petra Maresova, Lenka Komarkova, Jitka Kuhnova, Richard Cimler, Peter Pazitny, Daniela Kandilaki, Kamil Musilek, Zuzana Truhlarova, Filip Zemek, Kamil Kuca

**Affiliations:** 1 Department of Economics, Faculty of Informatics and Management, University of Hradec Kralove, Rokitanskeho 62, 50003 Hradec Kralove, Czech Republic; petra.maresova@uhk.cz; 2 Department of Exact Methods, Faculty of Management, Prague University of Economics and Business, Jarosovska 1117, 37701 Jindrichuv Hradec, Czech Republic; lenka.komarkova@vse.cz; 3 Centre of Advanced Technologies, Faculty of Science, University of Hradec Kralove, Rokitanskeho 62, 50003 Hradec Kralove, Czech Republic; jitka.kuhnova.2@uhk.cz (J.K.); richard.cimler@uhk.cz (R.C.); 4 Department of Management, Faculty of Management, Prague University of Economics and Business, Jarosovska 1117, 37701 Jindrichuv Hradec, Czech Republic; peter.pazitny@vse.cz (P.P.); daniela.kandilaki@vse.cz (D.K.); 5 Department of Chemistry, Centre of Advanced Technologies, Faculty of Science, University of Hradec Kralove, Rokitanskeho 62, 50003 Hradec Kralove, Czech Republic; kamil.musilek@uhk.cz; 6 Department of Special Pedagogy, Faculty of Education, University of Hradec Kralove, Rokitanskeho 62, 50003 Hradec Kralove, Czech Republic; zuzana.truhlarova@uhk.cz; 7 Biomedical Research Centre, University Hospital Hradec Kralove, Sokolska 581, 50005 Hradec Kralove, Czech Republic; zemek.filip@gmail.com

**Keywords:** Alzheimer’s disease, costs, prediction model, Czech Republic

## Abstract

Increasing life expectancy in modern society is undoubtedly due to improved healthcare, scientific advances in medicine, and the overall healthy lifestyle of the general population. However, this positive trend has led to an increase in the number of older people with a growing need for a sustainable system for the long-term care of this part of the population, which includes social and health services that are essential for a high quality of life. Longevity also brings challenges in the form of a polymorbid geriatric population that places financial pressure on healthcare systems. Regardless, one disease dominates the debate about financial sustainability due to the increasing numbers of people diagnosed, and that is Alzheimer’s disease (AD). The presented paper aims to demonstrate the economic burden of social and healthcare services. Data from two regions in the Czech Republic were selected to demonstrate the potential scope of the problem. The future costs connected with AD are calculated by a prediction model, which is based on a population model for predicting the number of people with AD between 2020 and 2070. Based on the presented data from the two regions in the Czech Republic and the prediction model, several trends emerged. There appears to be a significant difference in the annual direct costs per person diagnosed with AD depending on the region in which they reside. This may lead to a significant inequality of the services a person can acquire followed by subsequent social issues that can manifest as a lower quality of life. Furthermore, given the prediction of the growing AD population, the costs expressed in constant prices based on the year 2020 will increase almost threefold during the period 2020–2070. The predicted threefold increase will place additional financial pressure on all stakeholders responsible for social and healthcare services, as the current situation is already challenging.

## 1. Introduction

Many countries face the growing issue of an ageing population, which can be characterised as one of the major phenomena of this century [1]. Along with other European countries, the Czech Republic is one of these. According to data published by the Czech Statistical Office [2], as of 31 December 2019, seniors aged 65 and over (65+) made up one-fifth of the Czech population. In addition, the prediction for the share of seniors 65+ in the Czech population for 2030 is 22.3% and for 2050 reaches 28.6% [3]. While ageing is indeed a triumph of human progress, the phenomenon and its transition in society need to be responsibly managed [4]. The goal is not simply to increase life-expectancy but rather to increase the quality of life at its mature stage, since being in good health is essential not only to the individual but also to entire families, communities, and societies [5]. Initially, life-expectancy started increasing by virtue of control of infectious diseases, while later, by the treatment and management of lifestyle diseases, meaning chronic diseases resulting from unhealthy lifestyle, such as diabetes, obesity, or high blood pressure [6]. The last several decades have seen an immense progress in medical sciences and, above all, in the growing spread and accessibility of healthcare. This tendency needs to continue, along with promoting healthy and sustainable lifestyles, systematically supporting healthcare strategies and working towards bringing health and care to as many people as possible [5]. These goals belong among the priorities of the World Health Organisation (WHO) [6].

Despite the benefits of increasing life expectancy at a time of an increasing number of seniors, the need for a correctly set up system of long-term care (LTC) for the elderly, including both social and health services, is increasingly being discussed for the welfare of European Union (EU) countries [7]. There is also growing pressure for a sufficient number of formal caregivers, as well as places in residential facilities for the elderly [8]. In addition, Horecky and Prusa [9] point out that the availability of residential social services for the elderly in the Czech Republic is below the EU average and there is no systematic centrally managed plan for building further necessary capacities. In fact, in the field of health and social care, there are critical points that do not provide sufficient support for patients and their caregivers. A major difficulty is presented by the fact that health and social care in the Czech Republic are provided by two separate systems rather than a single integrated one, which makes it challenging to introduce a single comprehensive solution when it comes to providing care to an ageing population. In the Czech Republic, the burden of caregiving is chiefly borne by family members, with 80% of the elderly being taken care of by family caregivers. While this certainly benefits the patient, there are also adverse impacts on the informal caregiver’s quality of life, which should be taken into account [10].

Since dementia and AD are diseases associated with a higher age, it follows that with the ageing population, also the number of AD patients is increasing. According to the predictions of the Czech Alzheimer’s Society [11], we can expect a total of 183,000 patients with dementia in 2020 and a twofold increase in 2050, amounting to 383,000 AD patients. AD is the most prevalent dementia among the elderly [12]. Its progress usually remains hidden until the first signs of mild cognitive impairment (MCI), when it is already too late to stop or reverse the disease [13]. In addition, treatment is based on symptomatic drugs that ameliorate its symptoms but have minimal or no effects on the progress of the disease [14]. For these reasons, a person diagnosed with AD has only a perspective of a few years. Such people usually have to rely on their family and the health or social system to help them once they are no longer able to care for themselves. Furthermore, no breakthrough medicine or drug for AD can be expected in the near future [15].

The Czech Alzheimer’s Society and the Czech Statistical Office have published a report that provides forecasts of patient population development, as well as raising awareness of the unpreparedness of the Czech health and social system to accommodate the growing number of AD patients. There are currently a total of 21,000 beds in 341 senior homes and social care clinics available for dementia and AD patients [16]. As for health spending per capita in the Czech Republic, it has been on the increase since 2005 but still remains below the EU average [17].

On 3 February 2016, the Czech government accepted the “National Action Plan for Alzheimer’s disease and similar diseases”, which ran from 2016 to 2019. The plan had 14 aims and 28 objectives. The Ministry of Health plays a key role in its implementation, in cooperation with the Ministry of Labour and Social Affairs and the Ministry of Education, Youth, and Sports. The follow-up strategy and systematic implementation of measures so far is not a separate topic but is addressed as part of other health or social areas [18,19]. According to Matl et al. [11], the Czech Republic will need to triple its existing capacity for patients with AD to have a similar volume of services that correspond with the European average.

In light of the current COVID-19 pandemic, it should be stated that dementia itself does not increase the risk of incurring the disease; however, patients with dementia are more likely to neglect the necessary precautions, which means that this group of patients is ultimately highly vulnerable. The pandemic therefore increases the burden on the caregivers of such patients, whose cognitive impairment brings additional risks in everyday life, particularly in times of a pandemic situation [20]. At the outbreak of the pandemic in spring 2020, the “Alzheimer Europe position regarding the allocation of scarce medical resources for intensive care services during the COVID-19 pandemic” [21] was published and was joined by the Czech Republic as well.

Given the heavy impact of AD on society and the complexity of the problem, this paper focuses on two selected regions in the Czech Republic, “Kralovehradecky kraj” (KHK) and “Kraj Vysocina” (VYS), to illustrate the economic burden of health and social care for AD patients [11]. These particular regions were selected based on the fact that they have a higher share of AD patients than other Czech regions [22]. Specifically, the KHK Region experienced an approximately 20% increase in people aged 65+ between the years 2002 and 2017, which corresponds with an up to 40% increase in the economic load index. The situation in the VYS Region is very similar. In terms of the economic load index in the VYS Region, it is expected to increase by almost 100% by 2050, when the average age is predicted to be 49.9, in contrast to the current 41.3. In terms of the ageing population, the selected regions can be therefore considered as representative of the Czech Republic.

## 2. Theoretical Background

The issue of the economic burden of treatment and care for patients with AD is addressed because of the ageing population at international and global levels, as well as in many research studies [23,24,25,26]. The reason for analysing this issue is the inconsistent and often non-existent record of the number of patients suffering from AD and thus, the availability of only partial data on the costs of treatment and care.

The results reported by researchers who aim to calculate the economic burden are widely varied in connection to the economic level of the country and the health services provided for people suffering from AD. For instance, Schwarzkopf et al. [27] estimate the total medical and non-medical costs at EUR 9408 per client per year, while Reese et al. [28] arrive at a total of EUR 13,080 per client with AD, which takes into account all services and costs in Germany. The greatest proportion of total costs is constituted by LTC, which makes up 43% of the total. Michalowsky et al. [29] present a case study of Germany based on the country’s population forecasts and AD prevalence. They calculate that by 2060, the total societal cost for patients with dementia will amount to EUR 194 billion. Based on the identification of the factors involved in the societal and economic costs of patients with AD in community living arrangements, Dodel et al. [30] estimated the total costs for three European countries. The mean monthly costs per patient with AD were calculated at EUR 1881 for France; EUR 2016 for the UK; and EUR 2349 for Germany. The share of informal care costs in the total ranged from 50% to 61%. The calculations of both the social and economic burden are based on global estimates of the number of people with dementia, which are then broken down into individual countries. Therefore, the results may be misleading. Similarly, Wimo et al. [31] base their cost estimates for dementia patients on a combination of the United Nations’ prevalence prognosis forecast and Eurocode’s prevalence figures. More up-to-date estimates further suggest that both the number of people with dementia and the costs associated with their care are rising. It is estimated that the costs of dementia in Europe will rise by approximately 43% between 2008 and 2030, amounting to more than EUR 250 billion [31]. According to the data provided by the World Alzheimer Report [32], dementia costs in individual European regions in 2010 and 2015 were EUR 210.1 billion in Western Europe, EUR 14.3 billion in Eastern Europe, and EUR 14.2 billion in Central Europe. Whereas the total cost in Europe in 2010 was EUR 238.6 billion, the forecast for 2015 was EUR 301.2 billion [32]. For 2050, Maresova et al. [33] forecast EUR 343 billion for AD client care and treatment. The estimates may be biased due to a lack of reliable data on the prevalence and incidence of AD in Europe and elsewhere. 

The accuracy of the estimates also depends on the method used to calculate them. Broulikova et al. [34] use the Monte Carlo simulation method and simulate two scenarios of lifetime societal costs per AD client. One scenario considers a homogeneous cohort counting 100,000 patients who went undiagnosed or were diagnosed late and receive the usual care. The other hypothetical scenario considers an identical cohort of patients who were diagnosed early and received prompt treatment. Regarding data on the cognitive decline and survival probability for treated versus untreated AD patients, these are taken from foreign clinical studies. Numerous studies [24,35,36] rely on mathematical models to arrive at qualified estimates of the development of AD in populations. Keogh-Brown et al. [37] used a dynamic computable general equilibrium model applied to the Chinese economy to analyse the economic and non-economic impacts of AD concerning prevalence, morbidity and mortality for the period 2011 to 2050. Based on prediction models, a major AD increase is expected in China in the 2011–2050 period, which will have significant macroeconomic consequences. Suh [38] adapted an existing Markov model to the situation in South Korea to analyse possible outcomes over a five-year period and assess the cost-effectiveness of galantamine in AD treatment. This study takes a numerical approach and considers the various AD stages.

## 3. Methodology

### 3.1. Study Design

The purpose of this work is to calculate the economic burden of care for people with AD in two selected Czech regions (KHK, VYS). We focused on the costs of health and social care from the perspective of health insurance funds and regional governments to support their budgetary planning function. The horizon for modelling of the economic burden is 2070.

The future costs are calculated using the prediction model, which is based on two pillars:

- A population model for predicting the number of people with AD between 2020 and 2070;
- A cost model for calculating the economic burden between 2020 and 2070 based on the predicted number of people with AD from the population model.

The approach chosen for predicting the AD population was a computational model using the proportions of patients with AD and the probability approach for different stages. This approach for AD population prediction is described in more detail by Cimler et al. [39]. The prognosis of the economic burden in the follow-up cost model is expressed in prices from 2017 to 2019 with regard to the available data. Thus, future social and healthcare costs are expressed in constant 2020 prices and are not discounted.

Table 1 shows the numbers and share of people aged 65+ in 2020, 2030, 2050, and 2070 in the selected regions. An increase in numbers by 2050 can be observed. The significantly lower number of people aged 65 and over in 2070 compared to 2050 is related to the overall decline in the population of the Czech Republic, and thus, also in its individual regions. It can be observed that the relative share did not decrease particularly significantly.

### 3.2. Population Model and Data Sources

**Figure 1:**
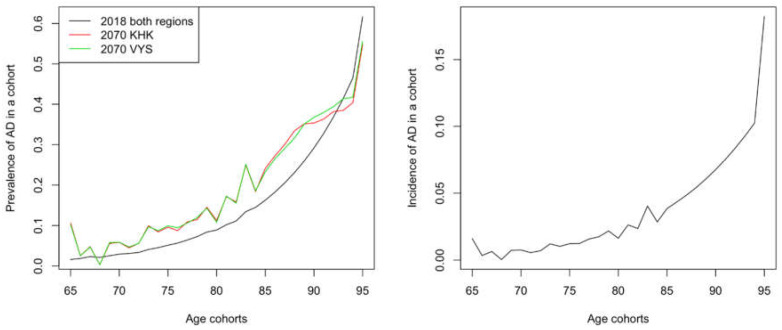
Prevalence in individual age cohorts at the beginning of the simulation (black line, same for both regions, the year 2018) and at the end of the simulation for each region. Source: authors’ own processing, 2020.

The initial data for the population model came from the Eurostat database [41]. There is a prediction of the development of each age cohort until 2100 for the Czech Republic. However, for individual regions in the Czech Republic, data from the Czech Statistical Office [40] are only publicly available with predictions until 2071. Therefore, the presented results based on the population model end that year for the selected regions. At the beginning of the simulation, the data on the Czech population were divided into two groups—a population with and without AD according to the determined prevalence by Tomaskova et al. [42]. Figure 1 shows the relationship of AD prevalence to age for the two selected regions—KHK and VYS.

In the next step in the simulation, prevalence is no longer used and incidence is used instead. The incidence was obtained from the baseline model using prevalence. Both the prevalence and the incidence parameters have a roughly exponential trend concerning age; see Figure 1. It is noticeable that in the case of incidence, there is an artefact for the cohort of 100-year-olds due to merging the people older than 100 from the Eurostat data into one group. Nevertheless, this artefact does not play a role in the simulation, as it is a very small group of patients who also “die” in the next step.

**Figure 2:**
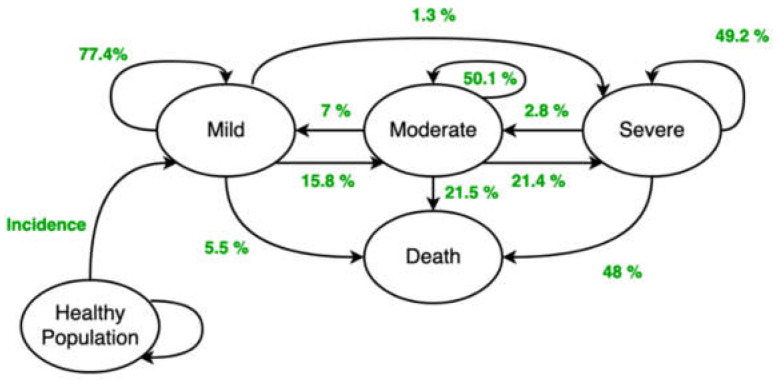
Theoretical model including quantified transition probabilities. Source: authors’ own processing, 2020, based on [43].

The initial selection of the AD population into individual stages is based on the limit distribution of these stages after the simulation that was run first. The prevalence of the disease was considered according to Tomaskova et al. [42] and during the first run of the programme, the corresponding incidences were found for individual age cohorts in each year of the simulation. Each additional simulation used the already determined incidences, which are independent of the choice of treatment scenario. The transitions between the Mild, Moderate, Severe, and Death stages (understood as a stage) are the closed “cycles” (Figure 2). The MCI stage is not currently considered in the basic version of the model but is planned for the future. As the last parameter, the proportions of the stages for individual age cohorts at the model initialisation had to be determined. These proportions were determined by convergence during the simulation.

An overview of all input variables and the subsequently supplemented values is presented in Table 2. The numbers of simulated people with AD are then linked to costs, which are determined in the next section.

### 3.3. Cost Model and Data Sources

The cost specification was monitored according to these cost groups [26]. Direct costs of AD are further divided into medical costs and non-medical costs. Medical costs cover drugs, medical material, diagnostic and monitoring tests, inpatient care, and outpatient care. Non-medical costs include costs for formal and informal care. Indirect costs, which often account for a significant proportion of the total costs (also in the context of the chosen country), calculate the loss of the productivity of patients and the productivity losses of their caregivers. This study focuses on direct medical and non-medical costs. Table 3 summarises the data sources used for the cost model, which are described in the following paragraphs.

Direct medical costs are calculated based on data obtained from the Institute of Health Information and Statistics of the Czech Republic (IHIS CR). These data contain expenditures on outpatient care, inpatient care, and medicines for 2017 in relation to stages of the disease (measured by MMSE) and age cohorts. The following variables were identified:

- Outpatient care—outpatient procedures administered by primary care medical doctors and specialists;
- Costs of medicines in outpatient clinics and hospitals (residential facility);
- Costs of hospital services;
- Hospitalisation—emergency care as well as other care.

Some costs were expressed in points that were converted into cash for modelling purposes. Table 4 shows the average direct medical costs per treated person for the KHK region and Table 5 for the VYS region. It is noticeable that outpatient care within the same age cohort is the most expensive at the mild stage of AD. The explanation may be that the health system intends to keep such patients in this disease stage for as long as possible, and, therefore, the treatment for such AD patients is more expensive than in other stages. These data from 2017 serve as inputs for the cost model—they constitute part of the direct medical costs.

Direct non-medical costs in our study are linked to social care services. In particular, the Service/Place Matrix described in one study [46] was used to identify the residential, outpatient, and domiciliary social services used by individual groups of people with AD. The prediction model takes into account the mild, moderate, and severe stages for AD patients. The findings based on the information obtained during interviews with representatives of the regional governments, nursing homes, and homes for the elderly were:

- People in the mild stage are mostly at home and are cared for by informal caregivers;
- Moderate-stage patients are also predominantly at home and use outpatient and domiciliary social services more often but there is a capacity problem, meaning many of them are unable to use these services;
- Patients suffering from the severe stage could be in residential social care facilities, but there is a capacity problem, meaning many of them cannot use these social services.

Data input into the model on capacities and prices/reimbursement in the social services sector were collected from open sources and verified by representatives of the KHK and VYS regions. Within residential services, the number of beds in the given facilities and their percentage use by patients with AD is taken into account. The share of AD patients using the services is based on a survey [45] and expert estimates. Specifically, in terms of residential services, the final calculation is tied to the price of a bed per day in the individual facilities (Table 6). For non-residential (outpatient and domiciliary) services, it is considered as a cost input of all full-time employees (FTE) that are allocated to a given service in the considered region and the percentage of these jobs that involve taking care of patients with AD (Table 7).

## 4. Results

### 4.1. Population of People with AD

A key issue in predicting the medical and non-medical costs of people with AD is to determine the number of persons suffering from this disease. Although there are data available on the number of diagnosed persons from IHIS CR, this is inaccurate and, according to all available information, also understated. Therefore, we could not rely on any convincing international study that would contain data for the Czech Republic. The prevalence of people with AD was therefore modelled.

**Figure 3:**
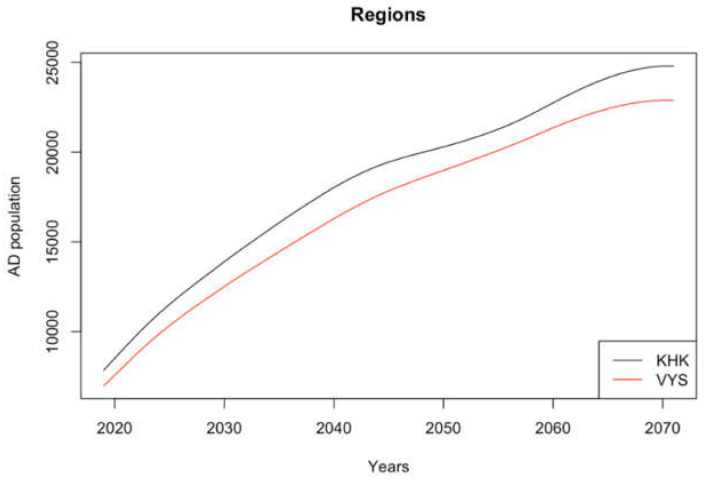
Development of the population of people with AD in the KHK and VYS regions. Source: authors’ own processing, 2020.

Figure 3 shows the number of people with AD in the selected regions as a result of the population model. The AD population can be expected to grow within the specified period, even between 2050 and 2070. The growth in both regions could be uniform and it is not expected that the proportion of patients with AD in one of the regions will significantly change. The prediction shows that the number of people with AD will increase up to threefold between 2018 and 2070.

### 4.2. Direct Costs

The costs of health and social care were modelled. Data were collected from different databases that are not interconnected so were, therefore, evaluated for each subsystem separately and consequently summarised. In the case of direct medical costs, it is assumed that not all people with AD receive treatment and not every treatment concerns only one patient with AD (a patient can be treated more than once during the year). On the other hand, it is expected that the regional proportion of treated patients with AD will remain the same during the simulation (there is no reason to believe that the proportion of treated patients with AD will increase or decrease). Therefore, the average cost per treated patient was calculated for each health cost category (Table 4 and Table 5). At the same time, the share of the total number of patients treated in 2017 was determined in relation to all people with AD according to regional prevalence and disease stage. These values remained constant throughout the simulation, and it was determined how many people with AD will be treated and how much the treatments will cost for each year.

For direct non-medical costs, it is assumed that the regional governments will supplement the capacity of social services together with the gradual increase in the number of people with AD in the individual disease stages. Therefore, for each year, the relative increase in the number of people with AD was determined. Along with this increase, the expected capacities in individual services while maintaining the unit financial costs were also increased.

Table 8 introduces the prediction model results. The development of costs in the two monitored regions shows the same trend. In both cases, the direct cost increased almost threefold over the period under review. The direct non-medical costs exceed direct medical costs. In the KHK region, this is more than four times; in the VYS region, this is more than eight times.

**Figure 4:**
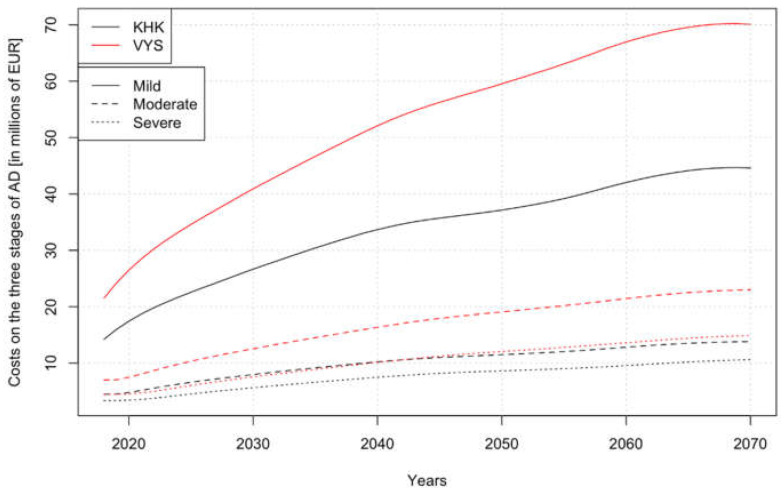
Direct costs in million EUR of the different disease stages in the selected regions. Source: authors’ own processing, 2020.

Figure 4 shows the economic burden for the expected development of the number of people with AD (displayed in Figure 3) in the future but at the current price level. Moreover, the total costs compared to Table 8 are related to the individual stages of the disease.

Specific costs per person with AD at a particular stage of the disease are shown in Table 9. This is the sum of both medical and non-medical (social) costs in the individual stages of the disease. Direct costs for the VYS region are significantly higher than for the KHK region due to higher social care unit costs (for a bed or FTE) and especially due to the higher capacities in special regime homes and daycare centres (see Table 6 and Table 7), although it is not possible to clearly specify patients according to the stage of the disease. Specifically, it is not possible to register data on the health condition of clients in social facilities, and therefore, the non-medical costs in Table 9 are the same for each disease stage.

## 5. Discussion

The results presented differ from the previously published studies in the approach to expressing non-medical costs, which in this case, are not directly tied to the particular person diagnosed with AD but are defined in relation to providing care at the provider level in the selected territorial unit (region) in the Czech Republic. Both current and predicted future costs are calculated assuming an ageing population. To acknowledge a certain bias, the total annual costs per person with AD were estimated at EUR 2812 for the KHK region (mild EUR 2689; moderate EUR 2628; severe EUR 3954) and EUR 4773 for the VYS region (mild EUR 4583; moderate EUR 4618; severe EUR 5900).

Our estimated values also correspond to a Czech study [47], where direct costs per client in a month were estimated to be EUR 243 (i.e., EUR 2916 per client in a year). There was a positive correlation between these costs and the increase in dementia severity. Compared to other countries, the Czech Republic manifests a lower price level per year. For example, Handels et al. [48] show the values in the Netherlands, where the mean total health care sector costs are EUR 26,140 per year and the mean total client and family costs are EUR 11,931. A German study [27] reports both medical and non-medical costs as EUR 9408 per year. Yet, another German study [28] suggests that the total cost per client is approximately EUR 13,080. 

However, all values focusing on the specification of the economic burden must be interpreted in the context that the person diagnosed with AD usually has additional physical and mental diagnoses [49] affecting their overall quality of life. The physical condition of the client may be affected by various accidental injuries, such as fractures incurred in falls or an increased risk of infection. The mental wellbeing of the client may suffer due to depression, psychosis, or agitation. These issues impact the societal and economic costs of patients with AD and, above all, also affect other family members. The state and its institutions should consider dementia as a complex problem and address it holistically. It is essential to start determining and calculating the total costs of dementia (medical and non-medical) to produce accurate data on which the social and economic policies will be based. This approach should also be adopted for other types of dementia or other diseases related to the ageing population.

The chosen approach has several limitations. The prediction model is based on the current state of treatment. For example, it does not take into account the prevention and early detection of new people with AD. Early diagnosis and treatment would increase the immediate medical costs due to the higher number of patients in the healthcare system. However, this could generate savings in the long term [34]. The data are collected from different databases that are not currently interconnected. Therefore, it is not possible to map the use of individual types of services at the level of individual patients. In other words, it is not possible to know from the presented data whether a given client receives health and social care at the same time, although the expenses for the average client for these two subsectors can be calculated. In the case of non-medical (social) costs, the costs per client are the same in each stage due to the absence of the necessary health data about clients of the Czech social system. Thus, it was not possible to distinguish the stages of AD. Further, direct medical costs are expressed in 2017 prices, while non-medical costs are expressed in 2019 or 2020 prices. Given the long period of time, everything is tied to the present and current prices. This shows a change concerning the changing AD population. In addition, the presented cost model does not include indirect costs that reflect the lost income of informal caregivers. Despite these limitations, the cost model in the form of a web application, which is currently under preparation, can be a useful tool to support managerial decision-making of the regional governments in the field of LTC for patients with AD. Based on this prediction model, they can plan the future capacity required to provide care for patients with AD, as well as the related future direct costs of the social and healthcare systems. In particular, the non-medical costs related to the social care system are important for all regional governments that contract and reimburse these services in the Czech Republic.

## 6. Conclusions

The authors of the presented work evaluated data from numerous official sources responsible for social and healthcare services concentrating on people diagnosed with AD and predicted how the current costs will evolve in the future for two regions of the Czech Republic. The available data were collected from institutions responsible for social and healthcare services and the model was devised to evaluate these separate datasets. The results were subsequently used to build a prediction model to determine how the medical and non-medical (social) costs will behave over time. It was found that care for people diagnosed with AD is provided in separate and, despite countless attempts, still unconnected departments in the health and social sectors. This is in line with previous research [50] that states (p. 80): “One of the key problems in the area of inpatient care in the Czech Republic is the separation of health and social care systems, both in terms of organisation and financing”. This has a negative effect on the oversight of the flow of money and the state and its institutions cannot plan adequate financial needs. As a result, there are promising activities in the VYS region to increase care integration and coordination of social and health services. Comparing the financial burden in both regions, direct costs for the VYS region are significantly higher than for the KHK region due to higher social care unit costs and especially due to the higher capacities in special regime homes and daycare centres. Both sectors evaluate the costs from only one perspective, social or medical, which does not provide the complex economic picture needed. As a result, the care for people diagnosed with AD is not correctly distributed and adequate, which means that family members have to put in additional financial and social resources. In this area, the VYS region tries to support informal caregivers by providing guidance, psychological support, and improved information flow. Not surprisingly, the number of AD patients and costs (medical and non-medical) connected to their care was found to increase over time and put the system under additional economic pressure, therefore, potentially decreasing the quality of life.

## Figures and Tables

**Table 1:** Ageing in the Kralovehradecky kraj (KHK) and Kraj Vysocina (VYS) regions.

Region	Number of 65+				Percentage of 65+			
	2020	2030	2050	2070	2020	2030	2050	2070
KHK	119,380	132,425	158,148	141,309	21.7	24.4	31.0	29.9
VYS	104,649	120,262	147,689	130,291	20.5	24.3	31.9	30.6

Source: Based on the Czech Statistical Office [40] data.

**Table 2:** Input variables in the population model for people with Alzheimer’s disease (AD).

Variable	Structure	Input Data	Initial Phase Description
Population size	Matrix 96 × 53 (96 age cohorts: 0–95+; 53 years: 2019–2071)	[40]	Population for regions from 2019 (the Czech Statistical Office data). For each region, there is a specific dataset with the same structure.
Prevalence AD	31 values (from 65 to 95+)	[42,44]	Based on the values for the EU, see Figure 1.
Incidence AD	31 values (from 65 to 95+)	[39]	Derived from AD prevalence, see Figure 1.
Transition probabilities	Matrix 4 × 4 (3 stages and death)	[43]	The transition probabilities between the disease stages (Figure 2).
Individual stage proportions	3 values (mild, moderate, severe)	Limit Distribution	Initial division of patients into individual stages.

Source: authors’ own processing, 2020.

**Table 3:** Overview of data sources for the cost model.

Costs	Data Source	Stage Identification	Year(s)
Medical	Institute of Health Information and Statistics of the Czech Republic	According to Mini Mental State Exam (MMSE) within age cohorts	2017
Non-medical	Webpages of the regional governments	Based on expert estimates from personal meetings	2019–2020
	Questionnaire [45] for social service providers		2019

Source: authors’ own processing, 2020.

**Table 4:** KHK region: Direct medical unit costs in EUR per treated patient with AD related to the stage and age cohort.

KHK		Outpatient			Inpatient		
Stage	Age Cohort	“Point” System	Other Services	Medicines	“Point” System	Acute Care	Other Care
Mild	65–69	435.77	31.77	72.00	0.00	243.42	442.54
	70–79	268.15	34.35	22.35	0.00	236.88	489.04
	80–89	187.77	15.23	33.35	0.00	264.69	574.23
	90+	164.85	6.27	3.35	0.00	263.15	976.08
Moderate	65–69	227.35	23.46	77.27	0.00	166.77	557.00
	70–79	212.31	24.12	9.46	0.00	291.65	772.62
	80–89	182.96	13.08	50.15	0.15	242.38	506.92
	90+	154.69	10.19	5.54	0.00	125.00	0.00
Severe	65–69	188.42	52.58	25.58	0.00	170.04	356.77
	70–79	213.77	21.85	294.54	0.00	307.88	685.92
	80–89	161.15	11.81	9.00	0.00	298.85	715.96
	90+	120.42	8.35	7.65	0.00	224.54	891.38

Source: authors’ own processing, 2020.

**Table 5:** VYS region: Direct medical unit costs in EUR per treated patient with AD related to the stage and age cohort.

VYS		Outpatient			Inpatient		
Stage	Age Cohort	“Point” System	Other Services	Medicines	“Point” System	Acute Care	Other Care
Mild	65–69	244.69	30.85	454.62	0.00	316.85	586.85
	70–79	232.23	19.54	75.58	0.00	194.00	453.85
	80–89	251.15	12.00	14.19	0.00	260.92	711.69
	90+	157.42	6.54	7.08	0.00	308.73	668.38
Moderate	65–69	194.35	24.38	1.85	0.00	107.42	761.92
	70–79	202.00	19.12	5.92	0.00	167.58	708.12
	80–89	201.35	8.65	3.92	0.00	193.62	722.12
	90+	187.04	13.50	4.54	0.00	263.73	868.12
Severe	65–69	185.35	25.23	2.73	0.00	187.19	647.42
	70–79	210.12	18.42	41.81	0.00	215.08	531.62
	80–89	261.31	9.08	12.58	0.00	257.73	832.12
	90+	157.92	3.23	19.58	0.00	276.35	987.19

Source: authors’ own processing, 2020.

**Table 6:** Number of beds in residential facilities, percentage utilisation by patients with AD, and unit costs (EUR per bed per day) of residential services.

Region	KHK			VYS		
Residential Services	Capacity (beds)	For AD (%)	Unit Cost (EUR)	Capacity (beds)	For AD (%)	Unit Cost (EUR)
Respite Care	67	78	44.65	39	78	71.15
Week Care Centres	9	47	45.58	0	47	76.92
Homes for the Elderly	2019	17	39.77	1977	20	51.92
Special Regime Homes	447	90	44.73	812	80	53.85
Health Care Facilities	34	47	23.77	57	47	33.08

Source: authors’ own processing, 2020.

**Table 7:** Number of full-time employees (FTE) in non-residential social services, percentage utilisation by patients with AD, and unit costs (EUR per FTE per month) of non-residential services.

Region	KHK			VYS		
Outpatient & Domiciliary Services	Capacity (FTE)	For AD (%)	Unit Cost (EUR)	Capacity (FTE)	For AD (%)	Unit Cost (EUR)
Personal Assistance	82	50	1605.96	83	50	1961.54
Day Services Centres	8	50	2184.31	23	50	2153.85
Daycare Centres	31	50	1681.62	117	50	2153.85
Domiciliary Service	412	50	1804.54	405	50	1961.54
Respite Care	0	78	0.00	25	78	2346.15

Source: authors’ own processing, 2020.

**Table 8:** Medical and non-medical (social) costs in million EUR.

Costs/Year	2020	2030	2040	2050	2060	2070
KHK Region	25.636	40.288	51.384	57.263	64.425	69.018
Medical	4.289	6.866	8.589	9.592	10.695	11.245
Non-medical	21.347	33.422	42.795	47.671	53.730	57.773
VYS Region	38.484	61.034	78.587	90.674	102.025	107.983
Medical	3.977	6.441	8.229	9.490	10.683	11.184
Non-medical	34.508	54.593	70.358	81.184	91.342	96.799

Source: authors’ own processing, 2020.

**Table 9:** Medical and non-medical (social) annual costs in EUR per person with AD in regions in regard to and regardless of the disease stage.

Stage	Mild		Moderate		Severe		No Resolution	
Direct Costs	KHK	VYS	KHK	VYS	KHK	VYS	KHK	VYS
Medical	359	354	298	389	1624	1671	482	504
Non-medical	2330	4229	2330	4229	2330	4229	2330	4229
Total	2689	4583	2628	4618	3954	5900	2812	4733

Source: authors’ own processing, 2020.
